# Influence of different short peripheral cannula materials on the incidence of phlebitis in intensive care units: A post-hoc analysis of the AMOR-VENUS study

**DOI:** 10.62838/jccm-2026-0012

**Published:** 2026-04-30

**Authors:** Yutaro Shinzato, Hideto Yasuda, Haruka Taira, Yuki Kishihara, Masahiro Kashiura, Takashi Moriya, Yuki Kotani, Natsuki Kondo, Kosuke Sekine, Nobuaki Shime, Keita Morikane

**Affiliations:** Jichi Medical University Saitama Medical Center, Saitama, Japan; Alliance for Vascular Access Teaching and Research, Griffith University, Nathan, Queensland, Australia; Department of Clinical Research Education and Training Unit, Keio University Hospital Clinical and Translational Research Centre (CTR), Tokyo, Japan; Kameda Medical Center, Kamogawa, Japan; Chiba Emergency and Psychiatric Medical Center, Chiba, Japan; Division of Clinical Laboratory and Infectious Diseases, Graduate School of Biomedical and Health Sciences, Hiroshima University, Hiroshima, Japan; Yamagata University Hospital, Yamagata, Japan

**Keywords:** cannula material stiffness, short peripheral cannula, critically ill, intensive care unit, phlebitis

## Abstract

**Aim of the study:**

Short peripheral cannula (SPC)-related phlebitis occurs in 7.5% of critically ill patients, and mechanical irritation from cannula materials is a risk factor. Softer polyurethane cannulas reportedly reduce phlebitis, but the incidence of phlebitis may vary depending on the type of polyurethane. Differences in cannula stiffness may also affect the incidence of phlebitis; however, this relationship is not well understood. This study analyzed intensive care unit (ICU) patient data to compare the incidence of phlebitis across different cannula products, focusing on polyurethane.

**Material and Methods:**

This is a post-hoc analysis of the AMOR-VENUS study that involved 23 ICUs in Japan. We included patients aged ≥ 18 years, who were admitted to the ICU with SPCs. The primary outcome was phlebitis, evaluated using hazard ratios (HRs) and 95% confidence intervals (CIs). Based on the market share and differences in synthesis, polyurethanes were categorized into PEU-Vialon^®^ (BD, USA), SuperCath^®^ (Medikit, Japan), and other polyurethanes; non-polyurethane materials were also analyzed. Multivariable marginal Cox regression analysis was performed using other polyurethanes as a reference.

**Results:**

In total, 1,355 patients and 3,429 SPCs were evaluated. Among polyurethane cannulas, 1,087 (33.5%) were PEU-Vialon^®^, 702 (21.6%) were SuperCath^®^, and 276 (8.5%) were other polyurethanes. Among non-polyurethane cannulas, 1,292 (39.8%) were ethylene tetrafluoroethylene (ETFE) cannulas, and 72 (2.2%) used other materials. The highest incidence of phlebitis was observed with SuperCath^®^ (13.1%). Multivariate analysis revealed an HR of 1.45 (95% CI 0.75–2.8, p = 0.21) for PEU-Vialon^®^, 2.60 (95% CI 1.35–5.00, p < 0.01) for SuperCath^®^, 2.29 (95% CI 1.19–4.42, p = 0.01) for ETFE, and 2.2 (95% CI 0.46–10.59, p = 0.32) for others.

**Conclusions:**

The incidence of phlebitis varied among polyurethane cannulas. Further research is warranted to determine the causes of these differences.

## Introduction

Short peripheral cannulas (SPCs) [1] are routinely inserted into most patients admitted to the intensive care unit (ICU). However, SPC insertion is associated with various adverse events, including hematoma, skin inflammation associated with drug leakage, and phlebitis [2]. According to a recently published study, the incidence of SPC-related phlebitis in the ICU can be as high as 24.7% (45/1,000 cannula-days) [3]. The high frequency of occurrence underscores the significance of these issues in ICU settings. Importantly, phlebitis can be regarded as a major complication because, even mild phlebitis can cause pain and anxiety, whereas severe phlebitis can cause skin necrosis and infective endocarditis[4,5,6].

Among the various factors that contribute to the occurrence of phlebitis, such as the type and dosage of administered drugs, mechanical irritation to the vessel wall is considered important, with cannula material being a contributing factor [7,8,9]. SPCs are predominantly made of materials such as polytetrafluoroethylene, polyethylene, silicone, and polyurethane; variations in the incidence of phlebitis have been reported for each material [10,11,12,13,14,15]. Among polyurethanes, a specified polyurethane known as PEU-Vialon^®^ (BD, Franklin Lakes, NJ, USA) is designed to be more flexible than standard polyurethane and has been reported to result in a reduced incidence of phlebitis compared to polytetrafluoroethylene (PTFE), also known as Teflon^®^ (Chemours, Wilmington, DE, USA)[10]. Additionally, a study has indicated variations in phlebitis incidence between polyurethane cannulas of the same type, such as Vygon^®^ (Vygon Group, Écouen, France) and PEU-Vialon^®^[12]. Although the incidence of phlebitis varies among different cannula materials, specific factors that contribute to this phenomenon remain unclear.

In this context, we hypothesize that cannula stiffness plays a crucial role in the development of phlebitis and varies across commercially available products. This hypothesis is supported by reports indicating that polyurethane cannulas—particularly specified types such as PEU-Vialon^®^—exhibit progressive softening along the vessel wall compared to those from other manufacturers. [10, 14] Differences in the ratio of rigid to flexible fibers among polyurethane cannulas may contribute to this variability in stiffness [16]. These stiffness differences may influence the degree of mechanical irritation to the vessel wall, potentially affecting phlebitis incidence [10, 17]. However, the relationship between cannula stiffness and phlebitis in various SPCs from different manufacturers remains unclear. To resolve this knowledge gap, we aimed to examine the clinical data of ICU patients with SPCs and perform a comparative analysis of phlebitis incidence between different cannula products, particularly among polyurethanes.

## Methods

### Study Design

This was a post hoc analysis of the AMOR-VENUS study, a prospective, multicenter cohort study conducted between January 1 and March 31, 2018 in 22 institutions and 23 ICUs in Japan.[18] Ethical review was waived for this secondary analysis. The original study was approved and registered (UMIN000028019). This study followed the STROBE guidelines (Supplementary Table 1) [19].

### Participants

The AMOR-VENUS dataset included patients aged ≥ 18 years admitted to the ICU with SPCs inserted during ICU admission. The detailed inclusion and exclusion criteria have been described previously.[3] The current study excluded patients with SPCs inserted outside the ICU, as the detailed information on drugs administered through the cannula is crucial for the analysis. Patients without cannula material information were also excluded.

### Data collection

The following data were collected from the dataset: patient characteristics (age, sex, height, weight, body mass index [BMI], Charlson Comorbidity Index [20], Acute Physiology and Chronic Health Evaluation [APACHE] II score [21], Simplified Acute Physiology Score II [22], Sequential Organ Failure Assessment [SOFA] score [23], ICU admission location, type and category of ICU admission, presence of sepsis at ICU admission, and use of mechanical ventilation), SPC characteristics (medical staff inserting the cannula, provision of standardized drug administration measures in the ICU, insertion site, cannula materials, cannula size, antiseptic solution before cannulization, use of ultrasonography, number of attempts before successful insertion, difficulties with the insertions, type of glove, type of dressing, any infection during cannula dwelling, and duration of cannula dwelling), drugs administered via SPCs during the ICU stay (ampicillin/sulbactam, dexmedetomidine, lipid emulsion, fentanyl, heparin, midazolam, nicardipine, and noradrenaline) [24], ICU mortality, and outcome of phlebitis. Phlebitis was defined using the Phlebitis Scale developed by the Infusion Nurses Society (see Supplementary Tables 2 and 3)[25]. Detailed information on its definition and evaluation methods has been provided in the AMOR-VENUS study and in the Supplementary Methods.

### Exposure

Various polyurethane cannulas exist, but previous studies have treated them as a single material. Some, such as PEU-Vialon^®^and SuperCath^®^, are marketed as more flexible “specified polyurethanes,” although their exact compositions are undisclosed. Even among these, phlebitis incidence varies [12]. We speculate that specific factors within polyurethane cannulas, including specified variants, could influence the incidence of phlebitis. Therefore, a detailed classification of the cannula materials may reveal different results regarding the risk of phlebitis. The composition of polyurethane is generally not disclosed; thus, considering its significant market share and varying degrees of polymerization [16], we differentiated polyurethane into specified polyurethanes (PEU-Vialon^®^and SuperCath^®^) and other polyurethane products. We further categorized the non-polyurethane materials as polyethylene, ethylene tetrafluoroethylene (ETFE), and other materials for analysis. None of the cannulas were of the integrated type [26,27,28].

### Outcome Measures

The primary outcome measure was phlebitis (see Supplementary Methods for details).

### Statistical analysis

The risk factors for phlebitis were analyzed using hazard ratios (HRs). Continuous variables were presented as means and standard deviations or as medians and interquartile ranges (IQRs) and analyzed using analysis of variance or the Kruskal–Wallis test. Categorical variables were presented as absolute counts and percentages (%) and analyzed using Fisher's exact test or Pearson's chi-square test.

This marginal Cox regression analysis was conducted to assess the association between the timing of phlebitis onset and presumed risk factors, accounting for the within-patient and within-institution correlations between cannulas. Considering the potential differences in phlebitis incidence among various polyurethanes, other polyurethanes were chosen as the reference group. In this model, the time of SPC insertion in the ICU was defined as time zero. The occurrence of phlebitis, removal of the SPC, or the time of ICU discharge if the patient left the ICU with the SPC still in place were considered as censors. The outcome was the time from cannula insertion to phlebitis onset, assessed in 4-hour intervals. Based on a previous study [24], the following presumed risk factors for phlebitis were extracted: patient characteristics (age, sex, BMI, and APACHE II score), type of admission to the ICU, SPC characteristics (provision of standardized drug administration measures in the ICU, medical staff inserting the cannula, insertion site, cannula materials, and cannula gauge), and drugs administered via SPCs during ICU stay (ampicillin/sulbactam, dexmedetomidine, fat, fentanyl, heparin, midazolam, nicardipine, and noradrenaline). BMI was categorized into three groups based on the World Health Organization classification for the Asian population as follows: ≤ 18.5, 18.6–25, and > 25 kg/m^2^ [29].

The drugs included in this model as binary data were based on a previous study [24] and were selected for the following reasons: (1) the top six drugs were administered more frequently than 5% in all SPCs, (2) the calculated p-value of phlebitis in the multivariate marginal Cox regression analysis of previous studies was less than 0.05, and (3) the drugs were clinically important. Drugs with very small sample sizes were excluded and a maximum of eight drugs were selected based on categories (1)–(3). Given that the missing data were randomly distributed, imputation was not performed, and only patients with complete data were included in the analysis. Effect estimates were described using HRs and 95% confidence intervals (CIs). Multivariable analysis was performed, adjusting for all potential confounding factors (Supplementary Table 4). All statistical analyses were performed using EZR version 1.38 (Saitama Medical Center, Jichi Medical University, Saitama, Japan) and SAS Studio (SAS Inc., Cary, NC, USA), and a two-sided p-value of <0.05 was considered statistically significant.

## Results

### Patient and Cannula Enrolment

**Fig. 1.**
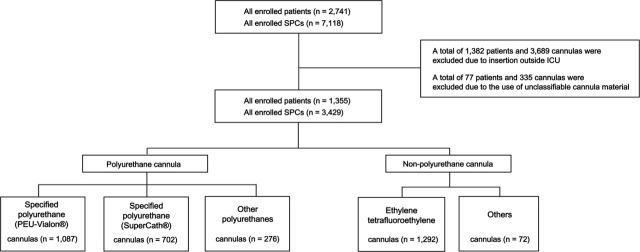
Patient inclusion flowchart.

In total, 2,741 patients and 7,118 SPCs were included in the analysis (Figure 1). Of these, 1,386 patients and 3,689 SPCs were excluded because of cannula insertion outside the ICU (n = 1,382 patients; 3,689 SPCs) or the use of unclassifiable cannula materials (n = 77 patients; 335 cannulas). Of the SPCs finally included, 1,087 (33.5%) specified polyurethane cannulas were PEU-Vialon^®^, 702 (21.6%) were SuperCath^®^, and 276 (8.5%) were made of other polyurethanes; there were 1,292 (39.8%) ETFE cannulas and 72 (2.2%) other cannulas.

### Cannula Characteristics

The patient characteristics and cannula materials are presented in Table 1. The highest incidence of phlebitis among polyurethane cannulas was observed with SuperCath**^®^** (13.1%). There were between-group differences between the cannula materials for all variables except age, sex, height, number of cannula insertions, and difficulty of insertion. The risk of septic shock was the highest with PEU-Vialon^®^ (173/1,087 cannulas [15.9%]). SuperCath^®^ demonstrated the highest rate of infection during cannula insertion (176/702 cannulas [25.1%]). Among the polyurethane cannulas, the longest duration of cannulization was 75.6 hours (IQR 84.7) for PEU-Vialon^®^.

**Table 1.** Comparison of the characteristics of short peripheral cannulas

**Variable**	**Cannula material** **Polyurethane**			**Non-polyurethane**		

	**Specified polyurethane**		**Other polyurethanes n=276 (8.5%)**	**Ethylene tetrafluoroethylene n=1,292 (39.8%)**	**Others n=72 (2.2%)**	**p-value**

	**PEU-Vialon^®^ n=1,087 (33.5%)**	**SuperCath^®^ n=702 (21.6%)**				
Phlebitis	83 (7.7)	92 (13.1)	17 (6.2)	111 (8.6)	10 (13.8)	< 0.01
Age (years)	67.4 [15.6]	69.0 [14.2]	67.3 [15.0]	68.2 [15.4]	64.3 [14.1]	0.03
Male sex	685 (63)	415 (59.1)	157 (56.9)	823 (63.7)	42 (58.3)	0.09
Body height^A^ (cm)	161.1 [9.9]	160.7 [9.3]	160.0 [10.2]	160.6 [9.5]	162.2 [9.7]	0.25
Body weight^B^ (kg)	60.0 [13.4]	62.0 [19.5]	59.2 [17.4]	59.1 [13.3]	63.6 [16.9]	< 0.01
BMI^B^	22.8 [4.1]	23.7 [5.78]	22.87 [4.68]	22.8 [4.21]	24.0 [4.87]	< 0.01
APACHE II score^C^	17.8 [7.6]	20.2 [8.7]	15.3 [6.0]	20.7 [8.6]	16.2 [7.4]	< 0.01
SAPS II score^C^	41.1 [18.3]	47.1 [19.5]	34.5 [15.5]	47.4 [19.7]	37.2 [18.6]	< 0.01
SOFA score ^C^	6.9 [4.0]	6.7 [3.5]	5.3 [3.2]	7.0 [3.5]	5.8 [3.7]	< 0.01
Charlson Comorbidity Index	4.4 [2.74]	4.1 [2.5]	3.7 [2.1]	4.2 [2.5]	3.6 [2.5]	< 0.01

ICU admission from						
Operating room	531 (48.6)	211 (30.1)	183 (66.3)	381 (29.5)	42 (58.3)	< 0.01
Emergency room	307 (28.2)	347 (49.4)	65 (23.6)	630 (48.8)	20 (27.8)	
General ward	223 (20.6)	45 (6.4)	28 (10.1)	201 (15.6)	8 (11.1)	
Outpatients	8 (0.7)	1 (0.1)	0 (0)	11 (0.9)	0 (0)	
Transfer from other hospital	18 (1.7)	98 (14.0)	0 (0)	69 (5.3)	2 (2.8)	

Type of admission to ICU						
Elective surgical	345 (31.7)	26 (3.7)	111 (40.2)	187 (14.5)	23 (31.9)	< 0.01
Emergency surgical	186 (17.1)	185 (26.4)	72 (26.1)	194 (15.0)	19 (26.4)	
Medical	556 (51.1)	491 (69.9)	93 (33.7)	911 (70.5)	30 (41.7)	

Sepsis at ICU admission						
Sepsis	60 (5.5)	46 (6.6)	7 (2.5)	156 (12.1)	0 (0)	< 0.01
Septic shock	173 (15.9)	63 (9.0)	12 (4.4)	177 (13.7)	10 (13.9)	

Medical staff inserting the cannula^a^						
Doctor	85 (10.2)	28 (5.0)	11 (6.5)	160 (14.7)	3 (15.0)	< 0.01
Nurse	749 (89.8)	536 (95.0)	160 (93.0)	931 (85.3)	17 (85.0)	
Medical technologist	0 (0)	0 (0)	1 (0.6)	0 (0)	0 (0)	
Provision of standardized drug administration measures in the ICU	1,067 (98.2)	702 (100.0)	276 (100.0)	1264 (97.8)	72(100.0)	< 0.01

Insertion site^b^						
Forearm	633 (58.8)	381 (54.7)	133 (48.2)	659 (51.2)	43 (66.2)	< 0.01
Upper arm	104 (9.7)	81 (11.6)	16 (5.8)	151 (11.7)	4 (6.2)	
Elbow	55 (5.1)	44 (6.3)	6 (2.2)	56 (4.4)	2 (3.1)	
Wrist	52 (4.8)	44 (6.3)	19 (6.9)	46 (3.6)	1 (1.5)	
Hand	134 (12.5)	82 (11.8)	91 (33.0)	190 (14.8)	10 (15.4)	
Lower leg	51 (4.7)	42 (6.0)	4 (1.5)	124 (9.6)	4 (6.2)	
Dorsal foot	46 (4.3)	22 (3.2)	7 (2.5)	61 (4.7)	1 (1.5)	

Cannula gauge^c^						
14G	0 (0)	0 (0)	0 (0)	1 (0.1)	0 (0)	< 0.01
16G	10 (2.0)	1 (1.2)	35 (12.7)	26 (2.0)	2 (3.4)	
18G	19 (1.8)	10 (1.5)	30 (10.9)	29 (2.3)	1 (1.7)	
20G	354 (33.3)	171 (24.6)	38 (13.8)	308 (24.1)	17 (28.8)	
22G	644 (60.5)	509 (73.3)	167 (60.7)	895 (70.1)	39 (66.1)	
24G	37 (3.5)	3 (0.4)	5 (1.8)	17 (1.3)	0 (0)	

Antiseptic solution before cannulization^d^						
None	1 (0.1)	1 (0.2)	1 (0.6)	5 (0.5)	0 (0)	
Alcohol	800 (96.5)	559 (99.1)	171 (98.8)	1049 (97/2)	20 (100)	
0.2% chlorhexidine alcohol	6 (0.7)	3 (0.5)	0 (0)	12 (1.1)	0 (0)	< 0.01
0.5% chlorhexidine alcohol	3 (0.4)	0 (0)	0 (0)	12 (1.1)	0 (0)	
1.0% chlorhexidine alcohol	15 (1.8)	1 (0.2)	1 (0.6)	0 (0)	0 (0)	
10% povidone iodine	1 (0.1)	0 (0)	0 (0)	1 (0.1)	0 (0)	
Other	3 (0.1)	0 (0)	0 (0)	0 (0)	0 (0)	
Use of ultrasonography^e^	35 (4.3)	5 (0.9)	4 (2.3)	14 (1.3)	0 (0)	< 0.01

Number of attempts for insertion^f^						0.72
1	642 (80.5)	447 (79.5)	138 (80.2)	876 (82.0)	16 (84.2)	
2	96 (12.0)	71 (12.6)	23 (13.4)	120 (11.2)	3 (15.8)	
3	33 (4.1)	34 (6.1)	9 (5.2)	54 (5.1)	0 (0)	
4	14 (1.8)	5 (0.9)	2 (1.2)	5 (0.5)	0 (0)	
5	6 (0.6)	1 (0.4)	0 (0)	8 (0.8)	0 (0)	
≧ 6	7 (0.9)	4 (0.7)	0 (0)	5 (0.5)	0 (0)	

Difficulties with the insertions^g^						
Easy	343 (44.1)	254 (45.1)	101 (58.7)	527 (49.4)	7 (43.8)	0.05
Slightly easy	248 (32.0)	186 (33.0)	41 (23.8)	294 (27.6)	3 (18.8)	
Slightly difficult	141 (18.2)	96 (17.1)	22 (12.8)	192 (18.0)	5 (31.3)	
Difficult	45 (5.8)	27 (4.8)	8 (4.7)	53 (5.0)	1 (6.3)	

Glove^h^						
Sterile	7 (0.9)	5 (0.9)	0 (0)	6 (0.6)	1 (5.3)	< 0.01
Non-sterile	743 (91.6)	521 (93.5)	170 (98.8)	1044 (97.5)	18 (94.7)	
None	61 (7.5)	31 (5.6)	2 (1.2)	21 (2.0)	0 (0)	

Dressing^i^						
Chlorhexidine-impregnated dressing	0 (0)	0 (0)	0 (0)	0 (0)	0 (0)	< 0.01
Sterile dressing	1037 (96.0)	697 (99.4)	271 (98.6)	1270 (98.8)	52 (94.6)	
Non-sterile dressing	34 (3.2)	4 (0.6)	4 (1.5)	15 (1.2)	3 (5.5)	
Gauze dressing	1 (0.1)	0 (0)	0 (0)	0 (0)	0 (0)	
Tape dressing	8 (0.3)	0 (0.3)	0 (0.2)	0 (0)	0 (0)	
Any infection during cannula dwell	225 (20.7)	176 (25.1)	25 (9.1)	367 (28.4)	10 (13.9)	< 0.01
Duration of cannula dwell (hours)	75.6[84.7]	63.7 [48.8]	54.3 [46.9]	56.7 [62.3]	65.6 [65.7]	< 0.01

Administered drug						
Ampicillin/sulbactam	44 (4.1)	57 (8.1)	8 (2.9)	88(6.8)	2(2.8)	< 0.01
Dexmedetomidine	83 (7.6)	74 (10.5)	27 (9.8)	97(7.5)	11(15.3)	0.024
Lipid emulsion	78 (7.2)	63 (9.0)	21 (7.6)	138(10.7)	8(11.1)	0.042
Fentanyl	100 (9.2)	72 (10.3)	44 (15.9)	236(18.3)	11(15.3)	< 0.01
Heparin	154 (14.2)	46 (6.6)	39 (14.1)	77(6.0)	18(25.0)	< 0.01
Midazolam	5 (0.5)	13 (1.9)	3 (1.1)	35(2.7)	4(5.6)	< 0.01
Nicardipine	82 (7.5)	113 (16.1)	37 (13.4)	68(5.3)	7(9.7)	< 0.01
Noradrenaline	28 (2.6)	17 (2.4)	4 (1.4)	40(3.1)	2(2.8)	-^**^

Missing patient data: A, n = 2 (0.2%); B, n = 4 (0.3%); and C, n = 90 (6.7%)

Missing data of cannulas: a, n = 748 (21.8%); b, n = 30 (0.9%); c, n = 61 (1.8%); d, n = 764 (22.3%); e, n = 793 (23.1%); f, n = 810 (23.6%); g, n = 835 (24.4%); h, n = 799 (23.3%); and i, n = 33 (1.0%)

Data are presented as n (%), median [interquartile range], or as the mean [standard deviation].

APACHE, Acute Physiology and Chronic Health Evaluation II; BMI, body mass index; ICU, intensive care unit; SAPS, Simplified Acute Physiology Score; SOFA, Sequential Organ Failure Assessment

PEU-Vialon^®^ is manufactured by BD, Franklin Lakes, NJ, USA; SuperCath^®^ is manufactured by Medikit Co., Ltd., Tokyo, Japan.

### Phlebitis Risk Factors by Cannula Material

The multivariate analysis results of all presumed risk factors as variables per cannula material type are summarized in Supplementary Table 4. Protective factors included the provision of standardized drug administration measures in the ICU (HR = 0.32, 95% CI 0.15–0.68, p < 0.01), cannula insertion by doctors (HR = 0.55, 95% CI 0.34–0.88, p = 0.01), and insertion at the upper arm (HR = 0.55, 95% CI 0.34–0.88, p = 0.01), all of which were associated with a reduced risk of phlebitis. In contrast, risk factors included the use of large-gauge cannulas (≤18G) (HR = 3.35, 95% CI 1.31–8.59, p = 0.01) and administration of nicardipine (HR = 1.79, 95% CI 1.26–2.54, p < 0.01), both of which significantly increased the incidence of phlebitis. Meanwhile, the multivariate analysis results of only the cannula materials as variables are shown in Table 2. Using other polyurethanes as references, the results showed that PEU-Vialon^®^ had an HR of 1.45 (95% CI 0.75–2.8, p = 0.21); SuperCath^®^ had an HR of 2.60 (95% CI 1.35–5.00, p < 0.01); ETFE had an HR of 2.29 (95% CI 1.19–4.42, p = 0.01); and other cannula materials had an HR of 2.20 (95% CI 0.46–10.59, p = 0.32).

**Table 2.** Multivariable analysis with marginal Cox regression analysis for the occurrence of phlebitis stratified by cannula material

**Variable**	**Multivariable analysis** **n = 3,429**	
	**HR (95% CI)**	**p-value**
Cannula material		
Polyurethane		
Specified polyurethane		
PEU-Vialon^®^	1.45 (0.75–2.8)	0.21
SuperCath^®^	2.60 (1.35–5.00)	< 0.01
Other polyurethanes	Ref	-
Non-polyurethane		
Ethylene tetrafluoroethylene	2.29 (1.19–4.42)	0.01
Others	2.20 (0.46–10.59)	0.32

CI, confidence interval; HR, hazard ratio; PEU-Vialon^®^ is manufactured by BD, Franklin Lakes, NJ, USA; SuperCath^®^ is manufactured by Medikit Co., Ltd., Tokyo, Japan.

## Discussion

This study showed that specified polyurethane cannulas, such as SuperCath^®^, and tetrafluoroethylene cannulas contributed to an increased incidence of phlebitis in the ICU. Furthermore, multivariate analysis revealed a difference in phlebitis incidence between PEU-Vialon^®^, and SuperCath^®^, suggesting that the incidence differs among polyurethane products. These results suggest that even among polyurethane cannulas, characteristics vary depending on the product. Thus, one cannot simply assume that polyurethane cannulas have a lower risk of phlebitis.

Phlebitis is an inflammation of the veins, primarily caused by chemical damage, thrombus formation, and physical irritation from the indwelling cannula. [30] Mechanical phlebitis results from factors such as the material, length, and gauge (thickness) of the cannula; insertion angle; securement leading to cannula movement; and irritation of the vascular wall [7,8,9]. Of these, mechanical irritation is profoundly influenced by properties of the cannula material, which can significantly impact vascular integrity [10, 17], Based on previous studies [10,11,12], we considered cannula material to be a key factor contributing to the incidence of phlebitis. Studies comparing PEU-Vialon^®^, a cannula made from specified polyurethane, and Teflon^®^have shown that PEU-Vialon^®^cannulas exhibit a lower incidence of phlebitis than those made from PTFE (Teflon^®^) for patients in various wards, including ICU, and in the perioperative period [11,14]. This difference is likely due to multiple factors, including PEU-Vialon^®^, s greater softness and smoother surface, which may help reduce mechanical irritation. Our study also found differences in the incidence of phlebitis when comparing PEU-Vialon^®^ with non-polyurethane materials. However, when comparing SuperCath^®^, the incidence of phlebitis was not necessarily lower than that of non-polyurethane materials.

The study by Gupta et al.[12] further supports this notion. In their study of 70 patients undergoing off-pump coronary artery bypass grafting, they observed significant variation in phlebitis incidence among different polyurethane cannulas. Vygon^®^, a newer generation polyurethane cannula, was associated with reduced inflammatory response, likely due to increased flexibility. In contrast, Vialon cannula (Insyte-W^®^; BD, Franklin Lakes, NJ, USA), a hybrid polyurethane copolymer coated with silicone elastomer, may cause more irritation. These results suggest that physical properties such as flexibility could significantly influence phlebitis risk. The variability in flexibility is plausible, considering that polyurethanes are synthesized by blending hard and soft segments [16]. Our findings, consistent with the study by Gupta et al.[12], underscore the importance of recognizing heterogeneity within polyurethane materials. Although the cannulas evaluated in their study (Vygon^®^) differ from those in ours (SuperCath^®^), the variation in phlebitis incidence among polyurethanes remains evident. Together, these findings emphasize the fact that the flexibility of the cannula may significantly influence the incidence of phlebitis. Optimizing cannula flexibility could minimize vascular irritation and prevent phlebitis. However, despite the insights gained from these studies, it remains unclear whether the stiffness of the cannula is associated with the occurrence of phlebitis.

To further explore the relationship between the stiffness of cannula materials and phlebitis, an additional study, independent of the main study, was conducted to investigate the stiffness of different cannula materials. This supplementary study specifically compared several types of polyurethane, as well as ETFE (Supplementary Figure 1). These findings showed significant differences in stiffness among the tested materials. Polyurethane materials become softer when exposed to warm water (similar to the temperature of blood), resulting in lower load values representing cannula stiffness compared to ETFE. Notably, even among polyurethanes, there were differences in stiffness. SuperCath^®^ exhibited the highest stiffness, which correlated with a higher incidence of phlebitis observed in clinical settings. While these results suggest a possible association between cannula stiffness and mechanical vascular irritation, this supplementary study was exploratory in nature. As stiffness and flexibility were not directly measured in the main study, causality cannot be established. Additionally, patient-related factors may also contribute to phlebitis development. The AMOR-VENUS study [24], which examined comorbidities and phlebitis risk without accounting for cannula type, provides further insights into patient-related factors and can be referred to as needed.

The present findings highlight the need for product-specific evaluation in cannula selection. Although all examined products were classified as polyurethane, substantial differences in phlebitis incidence were observed, suggesting that material classification alone is insufficient for risk assessment. Prioritizing cannulas with lower observed phlebitis rates may represent a more effective strategy. Optimizing mechanical properties—such as enhancing flexibility—may improve vascular compatibility. Further studies, including animal models and clinical trials, are warranted to validate these findings and inform the development of safer, patient-centered cannula designs.

This study has several limitations. First, it focused mainly on grade 1 phlebitis (73.8%), limiting generalizability to severe cases. Second, cannula stiffness was not assessed, restricting causal interpretation. Third, although key confounders were considered (e.g., patient characteristics, illness severity, procedures, drugs; Supplementary Table 4), unmeasured factors such as insertion techniques, staff experience, drug protocols, and dwell time may have influenced results. Standardized procedures are needed to minimize these effects. Finally, as this study was limited to ICU patients, generalization to non-ICU populations requires further validation.

## Conclusion

This study showed that the incidence of phlebitis varied among cannula materials, with SuperCath^®^showing the highest risk. These findings suggest that not all polyurethane cannulas offer the same safety profile, and material properties may affect phlebitis risk. Further research is warranted to confirm these findings and guide cannula design improvements.

## Abbreviations

ICU: intensive care unit;
SPC: short peripheral cannula
